# Ribosomal Protein Gene Knockdown Causes Developmental Defects in Zebrafish

**DOI:** 10.1371/journal.pone.0000037

**Published:** 2006-12-20

**Authors:** Tamayo Uechi, Yukari Nakajima, Akihiro Nakao, Hidetsugu Torihara, Anirban Chakraborty, Kunio Inoue, Naoya Kenmochi

**Affiliations:** 1 Frontier Science Research Center, University of Miyazaki Miyazaki, Japan; 2 Department of Biology, Graduate School of Science and Technology, Kobe University Kobe, Japan; Baylor College of Medicine, United States of America

## Abstract

The ribosomal proteins (RPs) form the majority of cellular proteins and are mandatory for cellular growth. RP genes have been linked, either directly or indirectly, to various diseases in humans. Mutations in RP genes are also associated with tissue-specific phenotypes, suggesting a possible role in organ development during early embryogenesis. However, it is not yet known how mutations in a particular RP gene result in specific cellular changes, or how RP genes might contribute to human diseases. The development of animal models with defects in RP genes will be essential for studying these questions. In this study, we knocked down 21 RP genes in zebrafish by using morpholino antisense oligos to inhibit their translation. Of these 21, knockdown of 19 RPs resulted in the development of morphants with obvious deformities. Although mutations in RP genes, like other housekeeping genes, would be expected to result in nonspecific developmental defects with widespread phenotypes, we found that knockdown of some RP genes resulted in phenotypes specific to each gene, with varying degrees of abnormality in the brain, body trunk, eyes, and ears at about 25 hours post fertilization. We focused further on the organogenesis of the brain. Each knocked-down gene that affected the morphogenesis of the brain produced a different pattern of abnormality. Among the 7 RP genes whose knockdown produced severe brain phenotypes, 3 human orthologs are located within chromosomal regions that have been linked to brain-associated diseases, suggesting a possible involvement of RP genes in brain or neurological diseases. The RP gene knockdown system developed in this study could be a powerful tool for studying the roles of ribosomes in human diseases.

## Introduction

Ribosomes are large ribonucleoprotein particles that catalyze messenger RNA-directed protein synthesis in all organisms. Eukaryotic ribosomes are composed of four ribosomal RNA (rRNA) species and about 79 different ribosomal proteins (RPs) [1], [2]. The higher-order structure of ribosomes has been well studied in prokaryotes using high-resolution crystallography, and the recent solution of co-crystals of ribosomes and other molecules, including tRNA, mRNA, and proteins, by cryogenic electron microscopy has improved our knowledge about the catalytic activities of ribosomes [3]. Although these findings demonstrated the central importance of rRNAs in ribosomes, and the structures of RPs and their interactions with RNAs have been thoroughly examined [4], the functions of RPs during translation have not been fully investigated. Quantitative deficiencies of RP genes have been suggested to contribute to the phenotype of the *Minute* mutants of *Drosophila*, which are characterized by delayed development; short, thin bristles; and recessive lethality; the RP gene deficiencies lead to growth retardation of the mutants by impairing the overall protein synthesis capacity of ribosome [5]. In fact, more than 50 *Minute* loci scattered throughout the genome have been found to encode RPs (S. Marygold, personal communication).

Unlike the widespread abnormalities of the *Minute* fly mutants, mutations in RP genes in mammals are associated with regional abnormalities. One example are the Tail-short (*Ts*) mutants of laboratory mouse strains; these mutants have short, kinky tails and numerous skeletal abnormalities. Heterozygosity for the *Ts* locus can be lethal, depending on the strain used for the cross, and the locus controlling this dominant lethality has been mapped [6]. Further studies revealed that *rpl38,* which is located within this locus, is altered in these mutants (T. Shiroishi, personal communication). Similarly, a deletion within *Rpl24* has been identified in mouse “Belly spot and tail” (*Bst*) mutants, which show a kinked tail, white hind feet, and a ventral midline spot [7]. For both *Rpl38* and *Rpl24*, expression of wild-type transgenes rescues the abnormal phenotypes (T. Shiroishi, personal communication) [7]. The only reported case of an RP gene mutation in human disease is Diamond-Blackfan anemia (DBA; OMIM 105650): *RPS19* is heterozygously mutated in 25% of unrelated patients with this disease [8], [9]. However, neither the candidate genes in the remaining 75% of the patients nor the role of RPS19 in erythropoiesis has been fully investigated. Therefore, the pathogenic mechanism of DBA remains unknown.

These reports suggest that RPs have unknown functions during organogenesis. The development of animal models will be essential for investigating these potential functions. Although *Rps19* knockout mice (*Rps19* ^−/−^) have been developed [10], they are not useful as a DBA model because *Rps19* ^−/−^ zygotes do not form blastocysts, whereas *Rps19* ^+/−^ mice show normal growth and organ development, including development of the hematopoietic system. It has been proposed that a regulatory mechanism compensates for the loss of one *Rps19* allele at the transcriptional or translational levels in the *Rps19* ^+/−^ phenotypes, resulting in the normalization of *Rps19* function [10]. Therefore, we hypothesized that the translational repression of RP mRNAs would be an effective approach for studying the functions of RPs during organ development. Zebrafish possess inherent advantages over other animal models because the embryos develop rapidly and the fish are easy to rear. Furthermore, morpholino antisense oligos (MOs) have been widely used in zebrafish to block the initiation of translation, and this method has proved to be highly reliable [11], [12]. In this study, we inhibited the translation of 20 RPs in zebrafish using MOs specific to each gene and examined morphogenesis in each of the embryos to investigate the usefulness of this system for developing models of RP-associated diseases.

## Results

### Development of RP Knockdown Fish Using MOs

A previous large-scale insertional mutagenesis screen in zebrafish identified more than 500 mutants, including mutants for several RP genes [13]. Therefore, the first step in our study was to examine whether the injection of MOs specifically targeting RP genes would result in embryonic phenotypes similar to those seen in insertional RP mutants. Of the RP genes identified in the insertional mutagenesis screen, we randomly selected the ribosomal protein L35 gene (*rpl35*) for the comparison and designed a specific MO for this gene to conduct a pilot injection study. Embryos injected with the MO against *rpl35* mRNA had small heads, round gray yolk sacs, and showed overall body degeneration, similar to what was described for the insertional mutant observed at 2 to 4 days post fertilization (dpf) [13]. Hence, our preliminary results suggested that use of MOs could be an easy and effective strategy to knock down RP genes. However, we noticed that the general phenotypic features observed with *rpl35* knockdown were common among the RP gene mutants at these later stages of embryonic development (2–4 dpf), when morphogenesis of primary organs systems is complete. We therefore chose to examine MO-injected embryos in more detail during the early stages of embryonic development, at 23.5–28.5 hours post injection when important organs such as brain, heart, and tail begin to develop. We designed specific MOs for 21 RP genes, chosen on the basis of previous studies suggesting their involvement in developmental process of some organisms, and carefully observed the morphants at about 25 hpf.

**Figure 1:**
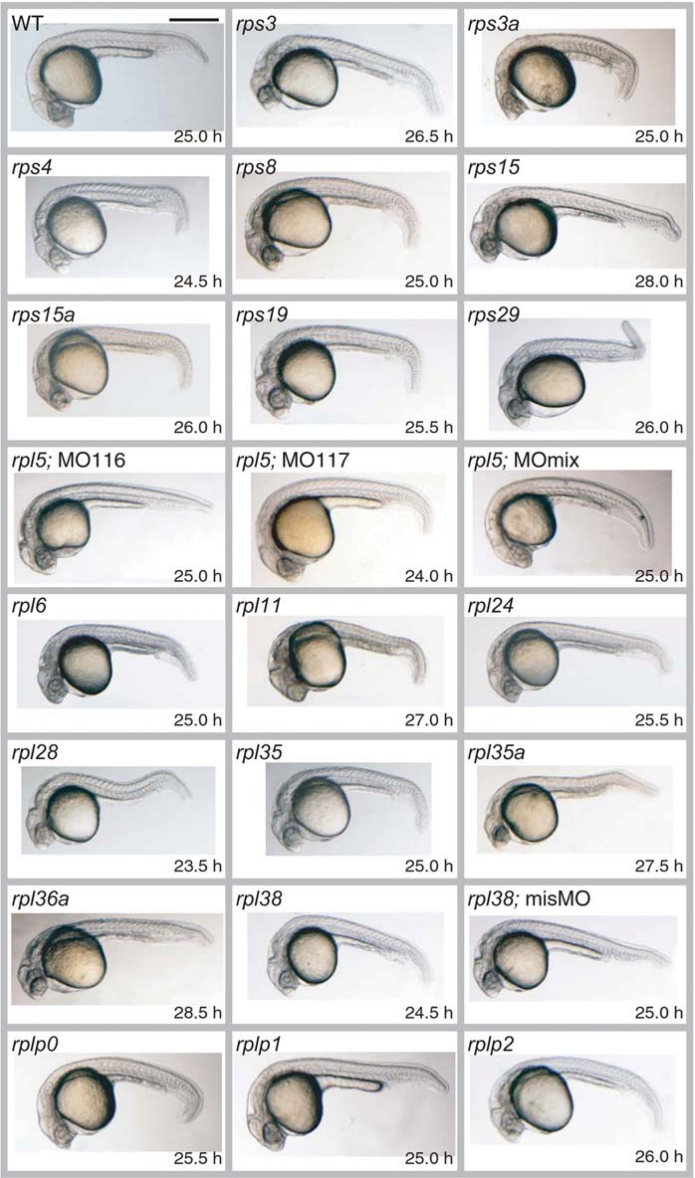
Lateral Views of Wild-Type and MO-Injected Embryos The genes targeted by MOs and the observation time are indicated for each image. ‘*rpl5*; MO116’ and ‘*rpl5*; MO117’ indicate two MOs designed and injected separately for two functional copies of *rpl5* on the zebrafish genome. ‘*rpl5*; MOmix’ indicates the mixture of MO116 and MO117. Control MOs that included 5 mispaired bases were also used; one example of a control MO injection is shown (*rpl38*; misMO). Note that compared to the control, the *rpl38* morphant is shorter and displays a light-colored eye and thin yolk sac extension. Scale bar, 500 µm.

We injected each MO into 20 to 50 fertilized eggs. The MO-injected embryos were compared to control embryos using a 24-point data sheet that included details regarding the shape, appearance, and morphology of various organs and overall condition of the embryos. The images and phenotypic descriptions of the embryos are available at the in-house database (http://zebrafish.med.miyazaki-u.ac.jp). We observed consistent phenotypes for each MO. A summary of common phenotypes observed among all the morphants at 23.5–28.5 hpf is presented in Table 1 and representative images of the morphants are shown in Figure 1. We also injected control MOs (misMOs) for the *rps4, rps19, rpl24, rpl35,* and *rpl38* genes (Table S1), and confirmed that the embryos did not display any morphological changes when compared to uninjected controls (see Figure 1, *rpl38*; misMO). We also confirmed that the abnormal phenotypes were rescued by injecting synthesized capped mRNA of *rpl38* (Figure S1). Hypoplasia of the yolk sac extension was evident in almost all the morphants except for *rpl5*; MO117 and *rplp1* (Figure 1). Cloudiness of the head region, delayed pigmentation in the retina, abnormalities in the ear, and reduction in melanophore pigments were observed in most of the morphants (Table 1 and Figure 1). Shortening of the body trunk was seen in the *rps3a, rps4, rps29, rpl6, rpl28, rpl35, rpl35a,* and *rpl38* morphants (Figure 1). Severely stagnant blood cells resulting from circulatory defects were observed in the *rps3a, rps4, rps15, rps15a, rps19, rps29, rpl6, rpl28, rpl35,* and *rplp0* morphants (Figure 1). All the embryos exhibiting abnormal phenotypes at 23.5–28.5 hpf died by 7–10 days post fertilization.

**Table 1:**
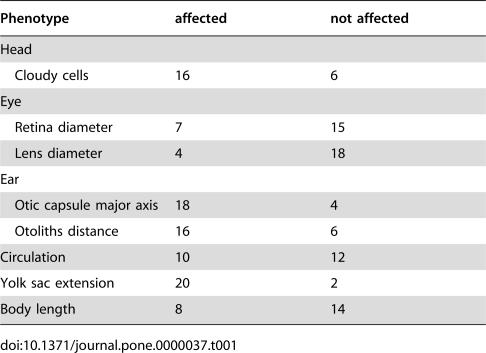
Summary of Phenotypes at About 25 hpf

Phenotype	affected	not affected
Head		
Cloudy cells	16	6
Eye		
Retina diameter	7	15
Lens diameter	4	18
Ear		
Otic capsule major axis	18	4
Otoliths distance	16	6
Circulation	10	12
Yolk sac extension	20	2
Body length	8	14

### Deformation of the Brain and Body Trunk

**Figure 2:**
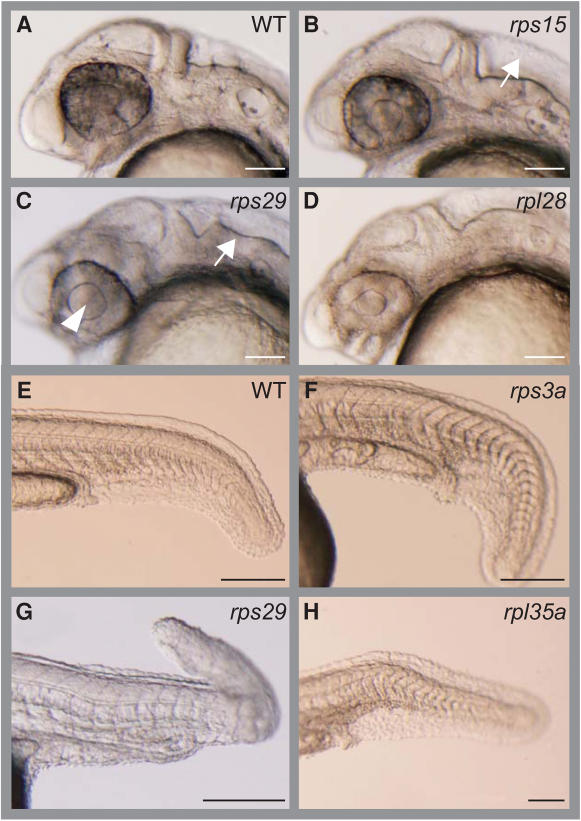
Specific Morphological Changes in Brain and Body Trunk Examples of morphants displaying characteristic deformations are shown. The targeted genes and the deformed areas of the morphants are indicated. (A, E) Wild type (WT). (B) *rps15*; enlarged 4th ventricle (white arrow). (C) *rps29*; enlarged lens (arrowhead) and undulated rhombencephalon (white arrow). (D) *rpl28*; protruded forehead. (F) *rps3a*; wider trunk and downward-curving tail. (G) *rps29*; wavy notochord and extremely bent tail. (H) *rpl35a*; sharply downward bent tail. Anterior is to the left. Bars: A∼D, 100 µm; E∼H, 200 µm.

The subdivisions of the developing brain can be identified under a stereoscopic microscope in zebrafish embryos at about 25 hpf. Abnormalities in the body trunk, including in circulation and notochord formation, can also be observed easily at this stage. The developmental defects in the brain and body trunk differed depending on the RP gene knocked down (Figure 2). For example, morphants of the *rps15*, *rps29,* and *rpl28* genes consistently showed an enlarged fourth ventricle (Figure 2B), undulated rhombencephalon (Figure 2C), and protruding forehead (Figure 2D), respectively. We also observed some distinct body trunk deformities, such as a curved tail (*rps3a,* Figure 2F), twisted tail (*rps29*, Figure 2G), or sharply bent tail (*rp135a*, Figure 2H).

**Figure 3:**
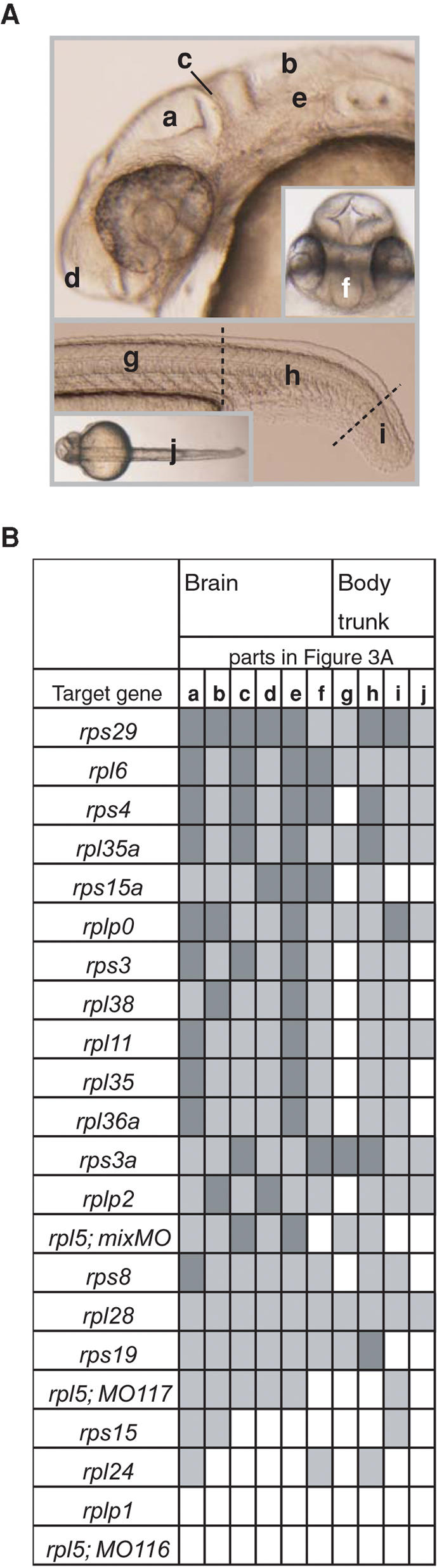
Schematic Representation of the Extent of Abnormalities in Brain and Body Trunk (A) 6 parts of the brain, 3 parts of the body trunk, and a dorsal view of the trunk are indicated in wild type embryo. a, optic tectum; b, 4th ventricle; c, midbrain-hindbrain boundary; d, anterior part of head; e, rhombencephalon; f, telencephalon; g, body along yolk sac extension; h, tail; i, tip of tail; j, dorsal view of the trunk. (B) The deformations were assessed using a 3-level severity scale; dark gray indicates a severe effect, light gray indicates a mild effect, and white represents no apparent effect. Detailed information and images of the abnormalities in the morphants are available at the zebrafish database (http://zebrafish.med.miyazaki-u.ac.jp).

To compare the brain and body trunk phenotypes in more detail, we scored the extent of abnormality in 6 parts of the brain, 3 parts of the body trunk, and the dorsal view of the trunk (Figure 3A) using a 3-level severity scale. The brain was most affected in the morphants of the *rps3, rps4, rps15a, rps29, rpl6, rpl35a,* and *rplp0* genes (Figure 3B). Within the brain, the telencephalon (Figure 3A-f) was much smaller than normal in morphants of *rps3a, rps4, rps15a,* and *rpl6*, whereas aplasia of the midbrain-hindbrain boundary (Figure 3A-c) was most prominent in morphants of *rps3, rps3a, rps4, rps29, rpl5mix, rpl6,* and *rpl35a*. The body trunk was extremely deformed in morphants of the *rps3a* and *rps29* genes. However, there was no correlation in the extent of deformity between the brain and the body trunk in a given morphant. For example, the brain was severely affected in *rps15a* morphants, whereas the tail was only mildly affected. Conversely, in the *rps3a* morphants, the brain exhibited only mild effects but the body trunk showed extreme deformities (Figure 3B).

### Disease Model

**Figure 4:**
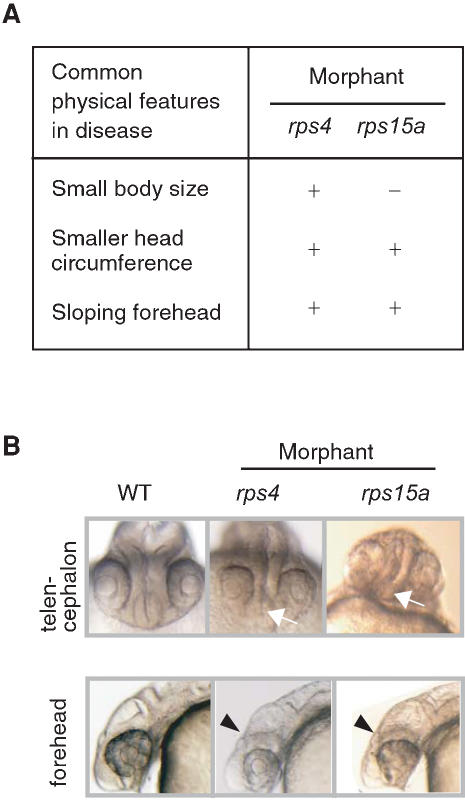
Similarities between the Clinical Features of Human Brain Diseases and Defects in Zebrafish Morphants (A) Common features shared between Abidi X-linked mental retardation and microhydranencephaly are listed on the left and the corresponding changes in *rps4* and *rps15a* morphants are indicated on the right. (B) Telencephalon hypoplasia is seen in *rps4* morphants, and reduction in size and deformity of the telencephalon is apparent in *rps15a* morphants (white arrows). Unclear subdivisions of the brain and flattened foreheads (arrowheads) can be seen in lateral views of these morphants.

We evaluated the possibility of using RP knockdown fish as models of human diseases. By searching the OMIM database (http://www.ncbi.nlm.nih.gov/entrez/query.fcgi?db = OMIM), we found that some human orthologs of the RP genes we targeted in zebrafish are located in chromosomal regions associated with diseases. Therefore, the phenotypes of our zebrafish knockdowns are likely to have some relevance to the clinical features of human diseases. Using the uniSTS database (http://www.ncbi.nlm.nih.gov/entrez/query.fcgi?db = unists), a comprehensive dataset of sequence tagged sites (STSs) that represent unique genomic positions on the human genome, we confirmed the positional relationships between the RP genes and the markers of the candidate chromosomal regions linked to disease. Our analysis revealed that the human *RPS4X*, *RPS15A*, and *RPLP0* genes are located in chromosomal regions linked to brain or neurological disorders (Table 2), and brain regions were affected in the morphants for these genes. Among the neurological diseases associated with the human genes, Abidi type X-linked mental retardation (XLMR; OMIM 300262) [14] and microhydranencephaly (MHAC; OMIM 605013) [15] share some common diagnostic features, such as shorter body, smaller head circumference, and sloping forehead (Figure 4A). In our study, the morphants of the *rps4* and *rps15a* genes, which may be related to these human diseases, also showed smaller head size and deformation of the telencephalon (Figure 4B), although these two morphants displayed otherwise different patterns of brain deformity (Figure 3B). The morphants of the *rplp0* gene, whose human ortholog is included in the candidate chromosomal region of Charcot-Marie-Tooth disease type 2L (CMT2L, a type of neuropathy; OMIM 608673), showed hypoplasia of the rhombencephalon that is strongly correlated with motor functioning (see the database: http://zebrafish.med.miyazaki-u.ac.jp).

**Table 2:**
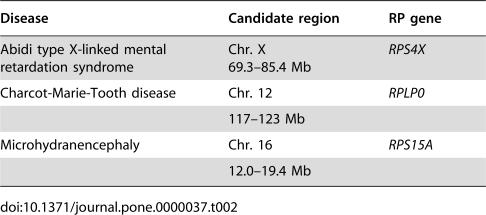
The Candidate Chromosomal Regions Linked to Brain Disease and the Human RP genes Included in These Regions

Disease	Candidate region	RP gene
Abidi type X-linked mental retardation syndrome	Chr. X69.3–85.4 Mb	*RPS4X*
Charcot-Marie-Tooth disease	Chr. 12	*RPLP0*
	117–123 Mb	
Microhydranencephaly	Chr. 16	*RPS15A*
	12.0–19.4 Mb	

## Discussion

### RP Knockdown System

In recent years, the relationship between ribosomal components or the ribosome itself and human diseases has received considerable attention. For example, defects in maturation of the 40S subunit or changes in level of RP gene expression may be associated with bone marrow failure and cancer susceptibility syndromes in humans [16], [17]. However, the present knowledge about this relationship is very limited and we have not yet understood completely whether defects in the ribosome are a cause or a consequence of such diseases. In our previous studies, we mapped and sequenced the human RP genes systematically [18], [19], and identified certain genes that might be involved in human disease based on a comparative analysis of their assigned genomic positions with candidate regions for Mendelian disorders [20]. To further understand this involvement, we needed to develop an appropriate vertebrate model for systematically studying the effects of ribosomal defects.

Here we developed a system in zebrafish using specifically designed MOs to inhibit the translation of RP genes. Out of a total of 79 RPs, we have so far developed morphants for 20 RPs. Some RP gene mutants were previously isolated from zebrafish using insertional mutagenesis [13], [21]. Although this insertional screening was carried out on a large scale aimed at identifying genes essential for early development, the phenotypic descriptions of these insertional mutants were rather preliminary and most of the images provided were taken at 2 to 4 dpf. In the current study, we observed the phenotypes of the MO-mediated RP knockdown embryos in more detail at 23.5–28.5 hpf and also examined the defects in particular organs, especially the brain.

Our results show that it is easy to develop zebrafish with RP defects that produce reproducible phenotypes using MOs as knockdown agents. Since the loss-of-function effect of injected MOs usually lasts for 2 to 4 days [12], such systems can be efficiently used to study how disruption of RP gene translation affects the early embryogenesis of vertebrates.

### Implications for the Study of Human Disease

Many genes encoding factors involved in ribosome synthesis are mutated in inherited bone marrow failure syndromes, including X-linked dyskeratosis congenita, cartilage-hair hypoplasia, DBA, and Shwachman-Diamond syndrome. In addition, several RP genes have been identified as tumor suppressor genes in zebrafish [22]. Although it is not clear whether the association between ribosomal defects and bone marrow failure is coincidental or causal, the various theories supporting this association have been examined in comprehensive reviews [16], [17].

In this study, we mainly focused on morphological changes in the brain regions of the morphants and on the possible association of ribosomes with brain diseases. Our results suggest that some RP genes might function in the morphogenesis of the zebrafish brain. Intriguingly, human orthologs of 3 RP genes, that affected the brain formation when knocked down, are included in the candidate regions of the brain and neurological disorders (Table 2). In humans, *RPS4X* is included in the chromosomal region that is a candidate location for the gene that causes Abidi type XLMR. Patients with the XLMR have short stature, small heads with sloping foreheads, and a broad range of somatic manifestations [14]. In this study, *rps4* morphants displayed severe deformities in the brain region, whereas the body trunk remained relatively unaffected (Figures 3B and 4B). Roughly 40% of the genes on the human X chromosome are expressed in the brain, suggesting their possible involvement in mental retardation, and to date 61 X-linked genes have been implicated in various types of XLMR [23]. Therefore, *RPS4X* could also play an important role in brain function. In CMT2L, an axonal neuropathy, the assigned locus contains 26 candidate genes, including the *RPLP0* gene. All the known exons and splice sites of these identified genes have been sequenced, and patient-specific mutations in *HSPB8*, which encodes the 22-kDa heat shock protein 8, were identified [24]. However, mutations in *HSPB8* have also been identified in patients with distal hereditary motor type II (dHMN II; OMIM 158590) disease [25], which is a third type of CMT with degradation of spinal cord anterior horn cells. Although it is still unknown how mutations in *HSPB8* cause these two different diseases, other genes may act synergistically with *HSPB8* to produce different symptoms. Genome mapping analysis has identified a locus for another type of Charcot-Marie-Tooth disease, CMT2C (OMIM 606071), 10 Mb upstream of the CMT2L locus, near the *RPL6* gene. In this study, the both *rplp0* and *rpl6* morphants had severe brain deformities (see the database: http://zebrafish.med.miyazaki-u.ac.jp and Figure 4B), indicating a possible relationship between these RPs and neurological disorders. The gene implicated in MHAC was localized to a minimal critical region of 7.4 Mb of the chromosome 16 [15], which includes the *RPS15A* gene. The phenotypic features of this disease are severe mental and motor retardation, small body size, and small occipital-frontal circumference without any other obvious abnormalities [15]. The *rps15a* knockdown zebrafish also showed striking features in the head, especially the telencephalon (Figure 4B), whereas the body trunk did not display any obvious abnormalities other than some mild effects on the tip of the tail (Figure 3B). Hence, we speculate that *RPS15A* might play a significant role in the pathogenicity of MHAC.

### Indication of Specific Functions of RP Genes

Large-scale chemical mutagenesis screens in zebrafish led to the identification of many mutants that displayed widespread, apparently non-tissue-specific abnormalities [26], [27]. It was assumed that mutations in cell-essential or housekeeping genes would have resulted in these general, frequently recurring mutants. Insertional mutagenesis screening, which, unlike chemical mutagenesis, enables efficient cloning of the disrupted genes, confirmed that mutations in genes required for essential cellular functions such as protein synthesis, RNA processing, DNA replication, and chromatin assembly often result in nonspecific developmental phenotypes. In contrast, lesions in genes encoding transcription factors, receptors, and ligands tend to result in mutants with specific developmental defects in one or a few organ systems [13].

Contrary to these findings, we found that inhibiting the translation of some RP genes, which are typically considered housekeeping genes, can give rise to specific changes in developing embryos (Figures 1 and 2). For example, the degree of abnormality within the brain subdivisions varied depending on which RP gene was targeted (Figure 3B). Moreover, when we repressed the expression of *rps19*, whose human ortholog is mutated in 25% of DBA patients [9], and performed hemoglobin staining at 48 hpf, the *rps19* morphants stained poorly when compared to other RP knockdown morphants, which could indicate a reduced red blood cell count (data not shown). Therefore, we speculate that the organs that display specific changes in response to RP knockdown may have a high sensitivity to a particular RP insufficiency, and this predictable sensitivity may be related to the specialized translational activity of ribosomes in different cells or organs.

Another example that suggests a specific role for a housekeeping gene is vanishing white matter disease (VWM; OMIM 603896). VWM is a chronic neurological disorder, and mutations in any one of the 5 genes encoding the subunits of eukaryotic translation initiation factor 2B (eIF2B) produce symptoms that are consistent with this disease. In most cases, the white matter of the brain, and particularly the glia cells, is affected, whereas the neurons are spared [28], [29]. Accordingly, it is conceivable that alterations in the expression of ubiquitous proteins such as RPs affect different cells or organs differently. Moreover, if RPs are critical in shaping the activity of the translational machinery, the activity of the ribosome would vary between cells. To address this possibility, a multifaceted approach is required, such as comprehensive analyses of gene expression and histological analyses of organs. The RP knockdown system developed in this study could be a valuable tool for such analyses. Considering the highly conserved nature of RP genes across vertebrate evolution, this knockdown system could also be instrumental in elucidating the pathogenic mechanisms of ribosomapathy.

## Materials and Methods

### Morpholinos

MOs were obtained from Gene Tools, LLC (Philomath, OR). The MOs were designed within 22 bp upstream and 22 bp downstream of the translation start site (AUG) for 21 RP genes: *rps3, rps3a, rps4, rps8, rps15, rps15a, rps19, rps29, rpl5* (2 copies), *rpl6, rpl11, rpl24, rpl28, rpl35, rpl35a, rpl36a, rpl38, rplp0, rplp1*, and *rplp2*. Since 2 functional copies of *rpl5* exist in the zebrafish genome, two MOs (MO116 and MO117) were designed separately for each copy. In addition, a mixture of these MOs was also used (*rpl5*; MOmix). Control MOs with 5 mispaired bases were also obtained for 5 RP genes: *rps4, rps19, rpl24, rpl35,* and *rpl38*. The sequences of the MOs are shown in Table S1.

### MO Injections

Zebrafish embryos at the one-cell stage were injected with the MOs using an IM-30 Electric Microinjector (NARISHIGE, Tokyo). Based on initial injection trials, 0.5 µg/µl (about 60 µM), was chosen as the optimal concentration for *rps3, rps3a, rps4, rps8, rps15, rps15a, rps19, rpl6, rpl11, rpl24, rpl35, rpl35a, rpl36a,* and *rpl38*, whereas 5.0 µg/µl (about 0.6 mM) was considered optimal for *rps29, rpl5* (both copies)*, rpl28, rplp0, rplp1,* and *rplp2*. Control MOs were injected at the optimal concentrations of their corresponding MOs.

### Observation of the Morphants and Database Construction

The MO-injected or uninjected embryos were grown at 28.5°C and observed for morphological changes under a stereoscopic microscope at 23.5 to 28.5 hpf. We assessed abnormalities in the shape, size, and morphology of the various organs in the morphants using an original 24-point data sheet (available at http://zebrafish.med.miyazaki-u.ac.jp). We also constructed a database to compare and record the morphological changes observed in the morphants for each RP gene. In addition to the dataset assessments, we further examined the morphants for specific abnormalities in 6 parts of the developing brain: optic tectum, fourth ventricle, midbrain-hindbrain boundary, anterior portion of the head, rhombencephalon, and telencephalon; 3 parts of the body: lateral trunk, midsection of the tail, and tip of the tail; and the dorsal view of the trunk. The extent of the deformities in these selected body parts was recorded using a 3-level severity scale. The eyes and ears of normal embryos ranged in size. For the injected embryos, if the measured size of the eye or ear was outside of the normal range, we considered these morphants to be affected by the knockdown. Body length was measured along the notochord from the posterior line of the otic capsule to the tip of the tail.

## Supporting Information

Table S1 The Sequences of the Morpholino Antisense Oligos Used in This Study (0.02 MB PDF)

Figure S1 Rescued Embryos Co-Injected with the MO for *rpl38* and Synthesized mRNA. Three days post fertilization embryos injected with the *rpl38*MO (0.5 µg/µl) displaying smaller head, shortened body and reduced yolk sac extension when compared to the wild type embryos. These phenotypes are rescued by co-injecting MO (0.5 µg/µl) and synthesized capped mRNA (0.5 µg/µl) for *rpl38* gene. The mRNA included altered bases that did not bind with the MO. Scale bar, 500 µm. The sequence information of the mRNA used for rescue is available at http://zebrafish.med.miyazaki-u.ac.jp.
(0.04 MB PDF)
