# Supplementary material for: Ribosomal Protein Gene Knockdown Causes Developmental Defects in Zebrafish
Source: PLoS One. 2006 Dec 20;1(1):e37. doi: 10.1371/journal.pone.0000037 (PMC1762390; doi:10.1371/journal.pone.0000037)
Supplement: Table S1 — (0.02 MB PDF) [file pone.0000037.s001.pdf]

Supporting Information

Table S1 – The Sequences of the Morpholino Antisense Oligos Used in This Study

Target gene	Morpholino oligo / mispaired oligo
rps3	CTTCTTCGAGATTTGCACCGCCATC
rps3a	TTTGCCGACTGCCATGTGAACAC
rps4	TGCTTCTTCGGTCCTCGGGCCATGT
	TGgTTCTTgGGTgCTCcGGtCATGT
rps8	TATCCCTTGAGATACCCATTCTC
rps15	CTTCTTCTGCTCAGTGTCCGCCATC
rps15a	CGCACCATGATGCCAGTTCTGCAAT
rps19	CACTGTTACACCACCTGGCATCTTG
	CACTcTTAgACgACCTGcCATgTTG
rps29	TCCAGTAGAGCTGCTGATGGCCCAT
rpl5	GACTTTTCAGTCTCCTAAGCCGGAG
	ACCCATTTTGTGATCGTTTGTTCTC
rpl6	CTTCTTCTTATCGCCCTCAGCCATC
rpl11	CTTCTTCTCGCTCTGGTCCGCCATG
rpl24	ACTGCACAGCTCGACCTTCATGGCG
	ACTcCAgAGCTCcACCTTgATcGCG
rpl28	CATTGCAGGTGAGGCGATGCCATGA
rpl35	GGTCTCTGGCCTTGATCTTTGCCAT
	GGTgTCTcGCCTTcATCTTaGCgAT
rpl35a	GGCATGATGATCCTTTGACCAGGCT
rpl36a	CATGGTTGCCCTCGCGGCGCAGGAG
rpl38	TTCTTCGATTTTACGTGGCATTGTG
	TTgTTaGATTTTAgGTGGgATTcTG
rplp0	CCTGTCTTCCCTGGGCATCTTTGCA
rplp1	AGGCGAGTTCGGACACAGATGCCAT
rplp2	GTAACGCATCTTTGCGGAGAGAAGG

‘CAT’ in red corresponds to the start codon of the targeted mRNA. Lower case letters indicate bases mismatched to the targeted mRNA.
