# Supplementary figures and images for: Ribosomal Protein Gene Knockdown Causes Developmental Defects in Zebrafish
Source: PLoS One. 2006 Dec 20;1(1):e37. doi: 10.1371/journal.pone.0000037 (PMC1762390; doi:10.1371/journal.pone.0000037)

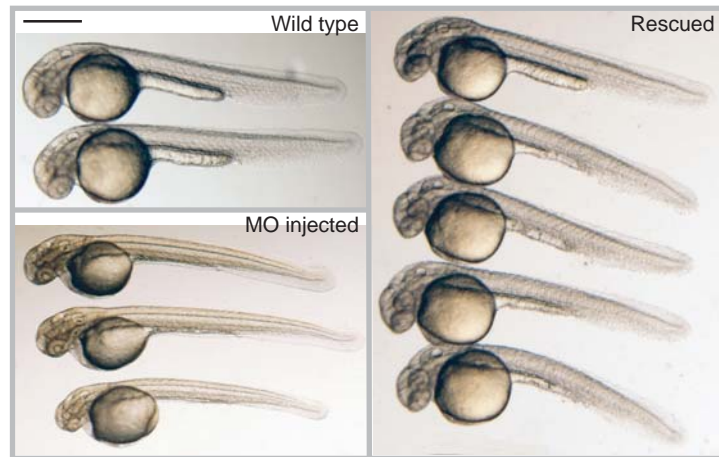

Figure S1\_UECHI

Supplement: Figure S1 — Rescued Embryos Co-Injected with the MO for rpl38 and Synthesized mRNA. Three days post fertilization embryos injected with the rpl38MO (0.5 µg/µl) displaying smaller head, shortened body and reduced yolk sac extension when compared to the wild type embryos. These phenotypes are rescued by co-injecting MO (0.5 µg/µl) and synthesized capped mRNA (0.5 µg/µl) for rpl38 gene. The mRNA included altered bases that did not bind with the MO. Scale bar, 500 µm. The sequence information of the mRNA used for rescue is available at http://zebrafish.med.miyazaki-u.ac.jp. (0.04 MB PDF) [file pone.0000037.s002.pdf]
